# Supplementary material for: Role and mechanism of NCAPD3 in promoting malignant behaviors in gastric cancer
Source: Front Pharmacol. 2024 Apr 22;15:1341039. doi: 10.3389/fphar.2024.1341039 (PMC11070777; doi:10.3389/fphar.2024.1341039)
Supplement: Supplementary file 11 [file DataSheet2.ZIP › GSEA/Transcipt factor/my_analysis.Gsea.1599461814734/BACH2_01.html]

Details for gene set BACH2\_01[GSEA]

|  || Dataset | filtered\_dataset.sample\_info.cls#WT\_versus\_NCAPD3\_MUT |
| Phenotype | sample\_info.cls#WT\_versus\_NCAPD3\_MUT |
| Upregulated in class | NCAPD3\_MUT |
| GeneSet | BACH2\_01 |
| Enrichment Score (ES) | -0.3812386 |
| Normalized Enrichment Score (NES) | -1.9996014 |
| Nominal p-value | 0.0074074073 |
| FDR q-value | 0.070614375 |
| FWER p-Value | 0.586 |
Table: GSEA Results Summary

  

Fig 1: Enrichment plot: BACH2\_01      
 Profile of the Running ES Score & Positions of GeneSet Members on the Rank Ordered List

  

| SYMBOL | TITLE | RANK IN GENE LIST | RANK METRIC SCORE | RUNNING ES | CORE ENRICHMENT || 1 | 7571 | ZNF23 | 25 | 1.018 | 0.0426 | No |
| 2 | 401474 | SAMD12 | 91 | 0.805 | 0.0437 | No |
| 3 | 23023 | TMCC1 | 181 | 0.695 | 0.0207 | No |
| 4 | 152485 | ZNF827 | 268 | 0.618 | -0.0046 | No |
| 5 | 1021 | CDK6 | 324 | 0.579 | -0.0098 | No |
| 6 | 23315 | SLC9A8 | 398 | 0.537 | -0.0306 | No |
| 7 | 571 | BACH1 | 884 | -0.300 | -0.3633 | Yes |
| 8 | 5292 | PIM1 | 885 | -0.300 | -0.3454 | Yes |
| 9 | 4616 | GADD45B | 904 | -0.330 | -0.3387 | Yes |
| 10 | 3164 | NR4A1 | 912 | -0.341 | -0.3234 | Yes |
| 11 | 23710 | GABARAPL1 | 913 | -0.341 | -0.3031 | Yes |
| 12 | 3691 | ITGB4 | 926 | -0.352 | -0.2908 | Yes |
| 13 | 6273 | S100A2 | 942 | -0.365 | -0.2799 | Yes |
| 14 | 9572 | NR1D1 | 977 | -0.392 | -0.2811 | Yes |
| 15 | 51646 | YPEL5 | 1014 | -0.413 | -0.2824 | Yes |
| 16 | 6016 | RIT1 | 1072 | -0.458 | -0.2963 | Yes |
| 17 | 3918 | LAMC2 | 1088 | -0.466 | -0.2793 | Yes |
| 18 | 8727 | CTNNAL1 | 1106 | -0.482 | -0.2629 | Yes |
| 19 | 639 | PRDM1 | 1159 | -0.523 | -0.2693 | Yes |
| 20 | 10221 | TRIB1 | 1229 | -0.604 | -0.2831 | Yes |
| 21 | 55 | ACPP | 1265 | -0.656 | -0.2693 | Yes |
| 22 | 84803 | GPAT3 | 1327 | -0.748 | -0.2688 | Yes |
| 23 | 1839 | HBEGF | 1328 | -0.751 | -0.2240 | Yes |
| 24 | 5997 | RGS2 | 1336 | -0.770 | -0.1831 | Yes |
| 25 | 1649 | DDIT3 | 1341 | -0.779 | -0.1395 | Yes |
| 26 | 255488 | RNF144B | 1362 | -0.834 | -0.1042 | Yes |
| 27 | 59277 | NTN4 | 1390 | -0.999 | -0.0641 | Yes |
| 28 | 4312 | MMP1 | 1407 | -1.304 | 0.0022 | Yes |
Table: GSEA details [plain text format]

  

Fig 2: BACH2\_01      
 Blue-Pink O' Gram in the Space of the Analyzed GeneSet

  

Fig 3: BACH2\_01: Random ES distribution      
 Gene set null distribution of ES for **BACH2\_01**
